# Social Mixing Patterns and Chikungunya Reemergence Risk in French Polynesia

**DOI:** 10.1001/jamanetworkopen.2026.2270

**Published:** 2026-03-18

**Authors:** Kiyoji-ken Chung, Maite Aubry, Iotefa Teiti, Mihiau Mapotoeke, Raihei White, Hervé Bossin, Françoise Mathieu-Daudé, Lisa Dian, Tuterarii Paoaafaite, André Wattiaux, Henri-Pierre Mallet, Jessica Vanhomwegen, Jean-Claude Manuguerra, Adam Kucharski, Van-Mai Cao-Lormeau

**Affiliations:** 1 Laboratory of Research on Emerging Viral Diseases, Institut Louis Malardé, Papeete, Tahiti, French Polynesia; 2 Unité des virus émergents, Aix-Marseille University, Università di Corsica, IRD 190, Inserm 1207, Institut de Recherche Biomédicale des Armées, Etablissement Français du Sang, Marseille, France; 3 Bureau de la veille sanitaire et de l’observation, Agence de Régulation de l’Action Sanitaire et Sociale, Papeete, Tahiti, French Polynesia; 4 Medical Entomology Laboratory, UMR 241 SECOPOL, Institut Louis Malardé, Paea, Tahiti, French Polynesia; 5 Environment and Infectious Risk Unit, Laboratory for Urgent Response to Biological Threats, Institut Pasteur, Paris, France; 6 Centre for Mathematical Modelling of Infectious Diseases, London School of Hygiene & Tropical Medicine, London, United Kingdom

## Abstract

**Importance:**

Chikungunya virus has reemerged in some French overseas territories and mainland France, raising concern for its reintroduction in French Polynesia.

**Objective:**

To assess current population immunity to chikungunya virus and estimate future outbreak risk.

**Design, Setting, and Participants:**

This cross-sectional study included data from 2 serosurveys and used a modeling approach that integrated seroprevalence, contact matrices, and demographic characteristics. The first serosurvey was conducted on the island of Tahiti (June 2018) among schoolchildren aged 6 to 16 years. The second serosurvey (November 2019 to December 2021) included adults aged 18 to 69 years across French Polynesia, a French overseas territory in the Southeast Pacific that comprises 75 inhabited islands grouped into 5 subdivisions with 279 000 people according to the 2022 census. Data analysis was conducted from April to June 2025.

**Main Outcomes and Measures:**

Main outcomes were seroprevalence rates and factors associated with seropositivity; age-stratified infection proportions for the 2014 to 2015 outbreak, pre-outbreak the basic reproduction number (R_0_), the effective reproduction number (R*_eff_*) for 2025, and R*_eff_* projections were estimated.

**Results:**

Serological data were available for 2363 participants (457 schoolchildren; median [IQR] age, 11 [8-13] years; 244 [53.3%] female, and 1906 adults; 38 [28-52] years; 1003 [52.6%] female). Seroprevalence was 62.8% (95% CI, 58.0%-67.0%) in schoolchildren, and the weighted seroprevalence was 67.6% (95% CI, 64.8%-70.3%) in adults. In adults, lower odds of seropositivity were associated with male sex (adjusted odds ratio [aOR], 0.71; 95% CI, 0.55-0.92), age groups 30 to 44 years and 45 to 69 years compared with 18 to 29 years (ages 30-44 years: aOR, 0.46; 95% CI, 0.32-0.66; ages 45-69 years: aOR, 0.33; 95% CI, 0.22-0.48), living in the Austral subdivision (aOR, 0.38; 95% CI, 0.27-0.55), and higher education (ie, university or more education: aOR, 0.51; 95% CI, 0.32-0.81). Living in larger households and being unpartnered were associated with higher odds of seropositivity (larger households: aOR, 1.69; 95% CI, 1.12-2.56; being unpartnered: aOR, 1.95; 95% CI, 1.03-3.69). Modeled infection rates for 2014 to 2015 ranged from 52.6% (95% credible interval [CrI], 43.6%-60.0%) in children aged 0 to 4 years to 80.8% (95% CrI, 73.3%-86.2%) in those aged 10 to 14 years. Inferred pre-outbreak R_0_ was 1.78 (95% CrI, 1.73-1.82), and R*_eff_* for 2025 was estimated at 0.95 (95% CrI, 0.85-1.04). Assuming assortative mixing, projections suggest R*_eff_* would exceed 1 (lower 95% CrI >1) by 2028, but assuming random mixing, R*_eff_* would exceed 1 in 2051, more than 20 years later than the other estimate.

**Conclusions and Relevance:**

In this cross-sectional study, high chikungunya seroprevalence was observed, with heterogeneity between subdivisions, age groups, and other sociodemographic characteristics. Modeling analysis estimated R*_eff_* near 1 in 2025, suggesting sustained transmission could occur if chikungunya is reintroduced. These findings suggest that incorporating contact matrices could improve outbreak risk assessments, while assuming random mixing might underestimate reemergence timing.

## Key Points

**Question:**

What is the risk of chikungunya reemergence in French Polynesia a decade after the first outbreak was recorded?

**Findings:**

In this cross-sectional study with seroprevalence data from 457 schoolchildren and 1906 adults, chikungunya seroprevalence among schoolchildren was 62.8% and weighted seroprevalence in adults was 67.6%. Modeling inferred a pre-outbreak basic reproduction number of 1.78 in 2014 to 2015, estimated an effective reproduction number (R*_eff_*) of 0.95 for 2025, and projected that the R*_eff_* would exceed 1 by 2028 assuming assortive social mixing.

**Meaning:**

The findings of this study suggest that despite high overall immunity, chikungunya transmission could be sustained following reintroduction; additionally considering age-specific contact matrices in risk assessment would help to refine estimated timing of reemergence.

## Teaser

This cross-sectional study examines the seroprevalence of chikungunya virus in French Polynesia and estimates the effective reproduction number for 2025 and later.

## Introduction

First isolated in East Africa in 1952, chikungunya virus (CHIKV) is an arthropod-borne virus (arbovirus) of the *Togaviridae* family.^1^ Its primary vectors are mosquitoes of the *Aedes* genus, particularly *Aedes albopictus* and *Aedes aegypti*. In French Polynesia, *Aedes polynesiensis* is also a competent vector.^2^ Common symptoms include high fever, headache, fatigue, maculopapular rash, severe polyarthralgia, and myalgia.^1,3^ It is estimated that between 3% and 25% of infections are asymptomatic.^4^ Additionally, approximately 40% to 80% of infected individuals may experience chronic symptoms (>3 months), particularly joint pain.^1,5^ No specific antiviral treatment is available. One live attenuated and one virus-like particle vaccine have been licensed with restrictions or suspended.^6,7,8,9,10^ Over the past 2 years, more than 600 000 cases per year have been reported worldwide, with model estimates suggesting millions each year.^6,11,12^

The recognition of CHIKV as a global public health concern emerged in the early 2000s. In 2006, La Réunion Island, a French overseas territory, experienced an extensive and severe chikungunya outbreak, along with other Indian Ocean countries.^13,14^ Subsequently, CHIKV caused multiple outbreaks in Latin America and the Caribbean, while circulating in Africa and Asia.^1,4,15^ In the Pacific, the first reported CHIKV infections occurred in New Caledonia (2011), followed by Papua New Guinea (2012), the Federated States of Micronesia (2013), Samoa (2014), and Fiji (2015).^16,17^ In French Polynesia, CHIKV was initially detected in May 2014 from a traveler returning from Guadeloupe (French Caribbean).^18,19^ Four months later, autochthonous cases were detected on Tahiti, and the virus subsequently spread throughout French Polynesia. While CHIKV was circulating in other Pacific Islands at that time, phylogenetic analyses revealed that French Polynesian strains were more closely related to those isolated in the Caribbean.^18^ By mid-March 2015, when the outbreak ended, an estimated 69 000 individuals (25% of the total population) had been affected, with 16 reported deaths.^20,21^ After the outbreak, a serosurvey conducted on the islands of Tahiti and Moorea revealed a CHIKV seroprevalence of 76%.^22^

In August 2024, CHIKV reemerged in La Réunion Island, causing more than 54 000 confirmed cases by June 2025, including 577 hospitalizations and 27 deaths.^23,24^ Considering the outbreaks in La Réunion Island and Mayotte, and locally acquired infections in metropolitan France,^24,25,26^ there was an increased risk of CHIKV reintroduction in French Polynesia.

This study describes chikungunya’s epidemiological status in French Polynesia and assesses the risk of a new outbreak. We conducted statistical analyses on data derived from 2 serosurveys: one involving schoolchildren on Tahiti in June 2018 and another involving adults across French Polynesia between November 2019 and December 2021. Moreover, as previous studies^27,28,29^ have highlighted the role of social structures and behaviors on arbovirus transmission, age-specific contact matrices were integrated into the modeling framework in addition to seroprevalence data.

## Methods

### Ethical Considerations

For this cross-sectional study, we used data from 2 serosurveys, one involving schoolchildren, which received approval from the Comité d’Ethique de la Polynésie française (CEPF), and the other involving adults, approved by both the CEPF and the French national Comité de protection des personnes. Written informed consent was obtained from all adult participants, and written parental consent was obtained for minors, who also provided verbal assent. This study followed the Strengthening the Reporting of Observational Studies in Epidemiology (STROBE) reporting guideline for cross-sectional studies.

### Setting and Study Populations

Located in the Southeast Pacific, French Polynesia is a French overseas territory of 121 islands (75 inhabited), grouped into 5 administrative subdivisions: Windward Islands, Leeward Islands, Marquesas Islands, Austral Islands, and Tuamotu-Gambier Islands, spanning an exclusive economic zone of more than 5 million square kilometers.^30,31^ Island types range from flat coral atolls to volcanic high islands, creating diverse ecological conditions. The population is approximately 279 000 inhabitants (per the 2022 census), with most residents living on Tahiti.^32^

#### Schoolchildren Serosurvey

Conducted in June 2018, the study aimed to assess the proportion of children aged 6 to 16 years immunized against arboviruses (eg, dengue, Zika, chikungunya, and Ross River viruses). Sociodemographic data and finger-prick blood samples were collected from 457 schoolchildren from 6 primary and secondary schools in Tahiti in June 2018.^22^ The survey was school-based with random selection of schools, classes, and schoolchildren among volunteers.

#### Adults Serosurvey (MATAEA Study)

Conducted in November 2019 to December 2021, this study’s main objective was to estimate the prevalence of both noncommunicable and communicable diseases as well as associated risk factors within the population.^34^ The study used a population-based sampling design to generate representative estimates for the adult population of French Polynesia.^34^ A total of 1942 participants aged 18 to 69 years were recruited. Participants completed a questionnaire that captured sociodemographic characteristics, lifestyle behaviors, and medical history.^34^ Physical measurements were recorded, and biological specimens (including blood) were collected. For both the schoolchildren and adult serosurveys, arbovirus IgG antibody detection, including those directed against CHIKV, was performed by microsphere immunoassay (MIA), as previously described.^33^

### Statistical Analysis

#### Data From Schoolchildren

Pearson χ^2^ tests were performed to assess the association between variables such as sex and age group. Weights were not applied to the data as sampling was restricted to Tahiti and complete information on selection probabilities was unavailable to derive valid design weights.

#### Data From Adults

##### Missing Data

Individuals without arbovirus MIA results were excluded from the analytical framework. For variables with missing percentages less than 1.6%, data were imputed using multiple imputation by chained equations, using random forest algorithm with a maximum of 5 iterations. The final sample size comprised 1906 individuals.

##### Weights

Weights were calculated as the ratio of the total population, stratified by subdivision, sex, and age group, to the number of observations in each corresponding category. Demographic data were obtained from the 2017 census.^34^ This weighting approach allowed the extrapolation of observations to the entire population while accounting for the demographic and geographic structure of French Polynesia.

#### Descriptive Analyses and Logistic Regressions

Pearson χ^2^ tests, adjusted using the Rao and Scott correction, were performed to assess the association between selected categorical variables. We performed weighted univariate logistic regression by including sociodemographic, environmental, and behavioral variables. All variables with a *P* value less than .20 were included in the initial weighted multivariable model. Subsequently, a manual stepwise backward elimination process was applied by successively removing non–statistically significant variables (*P* > .05) using the likelihood ratio test to compare successive models. The final variables retained were those that showed a statistically significant association with the outcome (*P* < .05). Statistical analyses were performed using R version 4.4.2 (R Project for Statistical Computing) with the gtsummary and survey packages for descriptive statistics and 95% CIs (weighted and unweighted), and the mice package for imputation.^35,36,37^

#### Models

##### Seroprevalence and Demographic Data

To simplify the dataset and retain only relevant variables for the modeling framework, we selected variables such as the participant identification number, sex, age, and confirmed serological status from the 2 serosurvey datasets, then added a new variable (ie, age at the time of the CHIKV outbreak). Data were then merged to obtain the main dataset. As the datasets contained no missing values for the selected variables, no imputation was required. Individuals were divided into 5-year age bins, from 0 to older than 75 years. Demographic data for French Polynesia were sourced from publicly available datasets for 2015 and 2024.^38,39^

##### Contact Matrices

Two types of age-stratified contact matrices were used. Empirical matrices, available via the socialmixr R package and derived from the Improving Public Health Policy in Europe Through the Modelling and Economic Evaluation of Interventions for the Control of Infectious Diseases (POLYMOD) study,^40,41^ which was conducted in 8 European countries (Belgium, Finland, Germany, Italy, Luxembourg, Netherlands, Poland, and the United Kingdom) and defined contacts as direct physical contact (eg, a handshake) or indirect contact (having a conversation of >3 words with another individual in the same place). Synthetic matrices were obtained from Prem et al,^42^ which provides social mixing estimates for 177 geographical regions through a modeling approach that integrates data from the POLYMOD study and multiple country-specific sociodemographic datasets. Here, we used the available data for France and 5 Pacific Island countries: Fiji, Samoa, Solomon Islands, Tonga, and Vanuatu.

##### Model Framework

To infer the basic reproduction number (R_0_; defined as the average number of secondary infections generated by a single infectious individual in an entirely susceptible population), we adapted the methods of Kucharski et al.^43^ We applied empirical and synthetic contact matrices to French Polynesia’s 2015 demographic structure. For each candidate R_0_, we used the equation
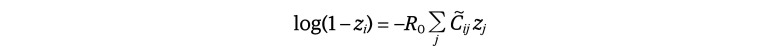
where *z_i_* and *z_j_* denote the final proportion of infected individuals in age groups *i* and *j*, respectively; *C̃_ij_*, the average number of effective contacts made by individual in age group *i* with age group *j*; and 1 − *z_i_*, the fraction of remaining susceptible individuals in age group *i*. Assuming complete susceptibility before the start of the outbreak in all age groups, we calculated the expected final proportion of infected individuals across all age groups (16 total) for the 2014 to 2015 outbreak by iteratively solving the final size equation (eFigure 1 and 2 in Supplement 1). We then inferred the R_0_ value that best fit observed seroprevalence data.

Next, we estimated the effective reproduction number (R*_eff_*; the average number of secondary cases generated per infectious individual in a population consisting of both susceptible and nonsusceptible individuals). Sustained transmission is expected when R*_eff_* is greater than 1, with an outbreak on average growing until it reaches its peak, at which point herd immunity is achieved. After this peak, additional infections still occur, but as the remaining proportion of susceptible individuals is no longer sufficient to sustain widespread transmission, the number of infections falls. Following such an outbreak, R*_eff_* is therefore substantially less than 1, but it gradually increases over time as new susceptible individuals are born or migrate into the population.

In the final part of our model framework, we estimated R*_eff_* in 2025, accounting for this gradual increase in susceptibility. To do so, we used French Polynesia’s demographic estimates in 2024, which represent the most recent population structure, and the same matrices used in the estimation of R_0_. We modeled heterogeneous susceptibility across age groups to reflect the population’s immune status following the 2014 to 2015 outbreak. We assumed full susceptibility for age groups younger than 10 years, which accounts for new births since the last outbreak. For each older age group, we progressively shifted susceptibility based on seroprevalence estimates, applying the same seroprevalence for the 2 oldest age groups due to unavailable data for these groups. We also estimated R*_eff_* assuming random mixing between age groups—as is common for models of vector-borne pathogens—for comparison. Simulations were performed using functions implemented in the finalsize R package.^44^

##### Parameter Estimation

We estimated R_0_ using bayesian inference with Markov chain Monte Carlo (MCMC) methods, as implemented in the MCMCpack R package.^45^ We ran 10 000 iterations with an initial burn-in of 1000 to ensure convergence and stable parameter estimates, with 95% credible intervals (CrIs) calculated from the 2.5th and 97.5th percentiles of the posterior distribution. We used a Bernoulli likelihood function that compared individual-level serostatus from the main dataset with age-specific probability of infection estimated by the model for the 2014 to 2015 outbreak. We calculated the effective reproduction number to evaluate the potential for future transmission. R*_eff_* was derived from our estimated R_0_ and the age-structured susceptibility of the Polynesian population in 2024. Specifically, R*_eff_* was computed using the formula *R_eff_ = R_0_ × ρ*(*S_0_*K),^46^ where S_0_ is a diagonal matrix of age-specific susceptibilities, and K is the next generation matrix.

##### Model Comparison and Sensitivity Analysis

Model comparison was performed by evaluating the maximized log-likelihood (log[L_MLE_]), and sensitivity analyses were conducted to assess the robustness of R_0_ estimates. Full details are provided in the eMethods and eFigures 3 and 4 in Supplement 1. Statistical analyses and modeling were performed in 2025.

## Results

### Schoolchildren Serosurvey

In Tahiti in June 2018, 457 schoolchildren aged 6 to 16 years (median [IQR] age, 11 [8-13] years; 244 [53.3%] female) were recruited for a serosurvey. Serological analyses revealed that 62.8% (95% CI, 58.2%-67.2%) were seropositive to CHIKV. Statistical analyses did not show a difference between sexes (female, 63.9% [95% CI, 57.5%-69.9%]; male, 61.5% [95% CI, 54.6%-68.0%]; *P* = .60). However, we observed that seroprevalence tended to increase nonlinearly with age, from 46.9% (95% CI, 38.1%-55.9%) in children aged 6 to 9 years to 72.6% (95% CI, 60.7%-82.1%) in those aged 14 to 16 years (Table 1).

**Table 1.** Seroprevalence of Chikungunya Virus in Schoolchildren in Tahiti in June 2018, Unweighted Analyses

Characteristic	Schoolchildren, No. (%) [95% CI]			Total, No. (n = 457)	*P* value^a^
	Seronegative (n = 170)	Seropositive (n = 287)			
Overall	170 (37.2) [32.8-41.8]		287 (62.8) [58.2-67.2]	457	NA
Sex					
Female	88 (36.1) [30.1-42.5]		156 (63.9) [57.5-69.9]	244	.59
Male	82 (38.5) [32.0-45.4]		131 (61.5) [54.6-68.0]	213	
Age, y					
6-9	68 (53.1) [44.1-61.9]		60 (46.9) [38.1-55.9]	128	<.001
9-14	82 (32.0) [26.4-38.2]		174 (68.0) [61.8-73.6]	256	
14-16	20 (27.4) [17.9-39.3]		53 (72.6) [60.7-82.1]	73	

Abbreviation: NA, not applicable.

^a^ Pearson χ^2^ test.

### Adult Serosurvey

Between November 2019 and December 2021, a total of 1942 adults were recruited for a serosurvey conducted across French Polynesia. Serological results were available for 1906 participants (median [IQR] age, 38 [28-52] years; 1003 [52.6%] female). We estimated a weighted CHIKV seroprevalence of 67.6% (95% CI, 64.8%-70.3%) (Table 2). We observed significantly higher CHIKV seroprevalence in female compared with male participants (female, 71.1% [95% CI, 67.2%-74.7%]; male. 64.2% [95% CI, 60.1%-68.1%]; *P* = .01). Seroprevalence decreased with age, from 80.8% (95% CI, 76.5%-84.5%) in those aged 18 to 29 years to 59.9% (95% CI, 54.9%-64.6%) in those aged 45 to 69 years. Our analyses also showed that CHIKV seroprevalence increased with household size, from 58.9% (95% CI, 52.4%-65.1%) in 1 to 2 person households to 76.4% (95% CI, 71.2%-81.0%) in households with 6 or more members, and varied by education level, ranging from 57.9% (95% CI, 51.6%-63.9%) for those with university or higher education to 72.9% (95% CI, 68.3%-77.0%) for those who completed high school. Seroprevalence also varied significantly by subdivisions, from 45.2% (95% CI, 38.7%-51.8%) in Austral Islands to 76.8% (95% CI, 70.6%-82.0%) in Tuamotu-Gambier Islands. However, seroprevalence did not differ significantly according to marital status, housing type, air conditioning and clean water access, self-reported mosquito-bite frequency, or number of mosquito-protection measures applied (Table 2).

**Table 2.** Weighted Seroprevalence of Chikungunya Virus in Adults in French Polynesia, 2019 to 2021

Characteristic	Participants, weighted % (95% CI)		*P* value^a^
	Seronegative (weighted n = 60 031)	Seropositive (weighted n = 125 111)	
Population estimates	32.4 (29.7-35.2)	67.6 (64.8-70.3)	NA
Sex			
Female	28.9 (25.3-32.8)	71.1 (67.2-74.7)	.01
Male	35.8 (31.9-39.9)	64.2 (60.1-68.1)	
Age group, y			
18-29	19.2 (15.5-23.5)	80.8 (76.5-84.5)	<.001
30-44	34.4 (29.8-39.3)	65.6 (60.7-70.2)	
45-69	40.1 (35.4-45.1)	59.9 (54.9-64.6)	
Highest education level			
End of primary school or before	32.9 (26.3-40.2)	67.1 (59.8-73.7)	<.001
End of secondary school	30.9 (25.8-36.4)	69.1 (63.6-74.2)	
End of high school or equivalent	27.1 (23.0-31.7)	72.9 (68.3-77.0)	
University or after	42.2 (36.1-48.5)	57.8 (51.5-63.9)	
Marital status			
Never married	29.0 (24.0-34.5)	71.0 (65.5-76.0)	.08
In a relationship	34.4 (31.1-37.8)	65.6 (62.2-68.9)	
Unpartnered	24.6 (16.2-35.5)	75.4 (64.5-83.8)	
Subdivision			
Windward Islands	33.5 (30.1-37.2)	66.5 (62.8-69.9)	<.001
Leeward Islands	26.5 (22.9-30.4)	73.5 (69.6-77.1)	
Marquesas Islands	30.5 (24.6-37.1)	69.5 (62.9-75.4)	
Tuamotu-Gambier Islands	23.2 (18.0-29.4)	76.8 (70.6-82.0)	
Austral Islands	54.8 (48.2-61.3)	45.2 (38.7-51.8)	
Household size			
1-2	41.2 (35.0-47.7)	58.8 (52.3-65.0)	<.001
3-5	33.6 (29.9-37.5)	66.4 (62.5-70.1)	
≥6	23.6 (19.0-28.8)	76.4 (71.2-81.0)	
House type			
House without garden	22.8 (14.7-33.6)	77.2 (66.4-85.3)	.06
House with garden	33.2 (30.4-36.1)	66.8 (63.9-69.6)	
Air conditioning			
Yes	35.6 (29.8-41.8)	64.4 (58.2-70.2)	.21
No	31.4 (28.4-34.5)	68.6 (65.5-71.6)	
Clean water access			
Yes	32.8 (30.0-35.7)	67.2 (64.3-70.0)	.21
No	25.4 (16.5-37.1)	74.6 (62.9-83.5)	
Mosquito bite frequency			
Never	26.6 (13.9-44.7)	73.4 (55.3-86.1)	.43
Rarely	35.5 (30.6-40.7)	64.5 (59.3-69.4)	
Often	31.8 (26.6-37.6)	68.2 (62.4-73.4)	
Every day	30.5 (26.6-34.7)	69.5 (65.3-73.4)	
No. of mosquito protections used			
0	31.3 (20.5-44.6)	68.7 (55.4-79.5)	.82
1-2	31.4 (27.3-35.9)	68.6 (64.1-72.7)	
3-4	32.5 (28.4-36.8)	67.5 (63.2-71.6)	
≥5	35.5 (28.2-43.6)	64.5 (56.4-71.8)	

Abbreviation: NA, not applicable.

^a^ Pearson χ^2^ with Rao and Scott adjustment.

Univariate regression yielded consistent results (Table 3), and these observations were largely consolidated by multivariate analysis. Males were less likely to be seropositive than females (adjusted odds ratio [aOR], 0.71; 95% CI, 0.55-0.92) (Table 3). Compared with participants aged 18 to 29 years, those aged 30 to 44 years and 45 to 69 years showed lower odds of being seropositive (30-44 years: aOR, 0.46; 95% CI, 0.32-0.66; 45-69 years: aOR, 0.33; 95% CI, 0.22-0.48). Individuals residing in the Austral Islands had lower odds of CHIKV seropositivity (aOR, 0.38; 95% CI, 0.27-0.55) than residents of the Windward Islands. In contrast, living in the Leeward Islands and the Tuamotu-Gambier Islands was associated with increased odds of seropositivity (Leeward: aOR, 1.33; 95% CI, 1.02-1.74; Tuamotu-Gambier: aOR, 1.68; 95% CI, 1.14-2.48). Living in a larger household (≥6 members) was also associated with increased odds of seropositivity (aOR, 1.69; 95% CI, 1.12-2.56). Additionally, individuals with higher education (ie, university or equivalent) had lower odds of seropositivity (aOR, 0.51; 95% CI, 0.32-0.81). Finally, unpartnered (separated, divorced, and widowed) individuals had higher odds of CHIKV seropositivity (aOR, 1.95; 95% CI, 1.03-3.69).

**Table 3.** Risk Factors Associated With Chikungunya Virus Seropositivity in the Univariate and Multivariable Logistic Regression Models

Characteristic	Univariate model			Multivariate model^a^		
	OR (95%CI)	*P* value	Global *P* value	aOR (95%CI)	*P* value	Global *P* value
Sex		.				
Female	1 [Reference]	NA	.01	1 [Reference]	NA	.01
Male	0.73 (0.56-0.94)	.01		0.71 (0.55-0.92)	.01	
Age group, y						
18-29	1 [Reference]	NA	<.001	1 [Reference]	NA	<.001
30-44	0.45 (0.32-0.63)	<.001		0.46 (0.32-0.66)	<.001	
45-69	0.35 (0.25-0.49)	<.001		0.33 (0.22-0.48)	<.001	
Highest education level						
End of primary school or before	1 [Reference]	NA	.001	1 [Reference]	NA	.002
End of secondary school	1.10 (0.73-1.64)	.65		0.93 (0.61-1.43)	.75	
End of high school or equivalent	1.32 (0.90-1.94)	.16		0.96 (0.63-1.48)	.86	
University or after	0.67 (0.45-1.01)	.05		0.51 (0.32-0.81)	.004	
Marital status						
Never married	1 [Reference]	NA	.09	1 [Reference]	NA	.04
In a relationship	0.78 (0.58-1.05)	.10		0.91 (0.65-1.27)	.55	
Unpartnered	1.25 (0.70-2.23)	.45		1.95 (1.03-3.69)	.04	
Subdivision						
Windward Islands	1 [Reference]	NA	<.001	1 [Reference]	NA	<.001
Leeward Islands	1.40 (1.09-1.80)	.009		1.33 (1.02-1.74)	.04	
Marquesas Islands	1.15 (0.82-1.61)	.41		1.22 (0.85-1.76)	.27	
Tuamotu-Gambier Islands	1.67 (1.17-2.38)	.005		1.68 (1.14-2.48)	.009	
Austral Islands	0.42 (0.31-0.57)	<.001		0.38 (0.27-0.55)	<.001	
Household size						
1-2	1 [Reference]	NA	<.001	1 [Reference]	NA	.04
3-5	1.38 (1.01-1.89)	.04		1.17 (0.83-1.65)	.40	
≥6	2.27 (1.55-3.32)	<.001		1.69 (1.12-2.56)	.01	
House type						
House without garden	1 [Reference]	NA	.07	NA	NA	NA
House with garden	0.60 (0.34-1.03)	.07		NA	NA	
Air conditioning						
Yes	1 [Reference]	NA	.21	NA	NA	NA
No	1.21 (0.90-1.63)	.21		NA	NA	
Clean water access						
Yes	1 [Reference]	NA	.21	NA	NA	NA
No	1.43 (0.82-2.51)	.21		NA	NA	
Mosquito bites frequency						
Never	1 [Reference]	NA	.43	NA	NA	NA
Rarely	0.66 (0.29-1.52)	.32		NA	NA	
Often	0.77 (0.33-1.80)	.55		NA	NA	
Every day	0.82 (0.36-1.89)	.64		NA	NA	
No. of mosquito protections used						
0	1 [Reference]	NA	.83	NA	NA	NA
1-2	0.99 (0.54-1.82)	.98		NA	NA	
3-4	0.95 (0.52-1.73)	.85		NA	NA	
≥5	0.83 (0.43-1.60)	.57		NA	NA	

Abbreviations: aOR, adjusted odds ratio; NA, not applicable; OR, odds ratio.

^a^ Nonsignificant variables (*P* > .05) were sequentially removed from the multivariate model using manual backward elimination. Results of the model without elimination are shown in eTable 3 in Supplement 1.

### Model Framework

#### R_0_ and Model Comparison

R_0_ was inferred using contact matrices from 14 countries (eTable 1 in Supplement 1). The matrices were categorized into 2 groups: 8 empirical matrices from European countries and 6 synthetic matrices derived from France and 5 Pacific Island countries. R_0_ estimates varied across matrices, ranging from 1.71 (95% CrI, 1.67-1.75) to 1.88 (95% CrI, 1.84-1.94). The model using the synthetic matrix of France yielded the highest log(*L_MLE_*) value (log[*L_MLE_*] = −1472), indicating the best fit to the seroprevalence data of French Polynesia, and estimated a pre-outbreak R_0_ value of 1.78 (95% CrI, 1.73-1.82).

#### Final Size Calculations

**Figure 1.**
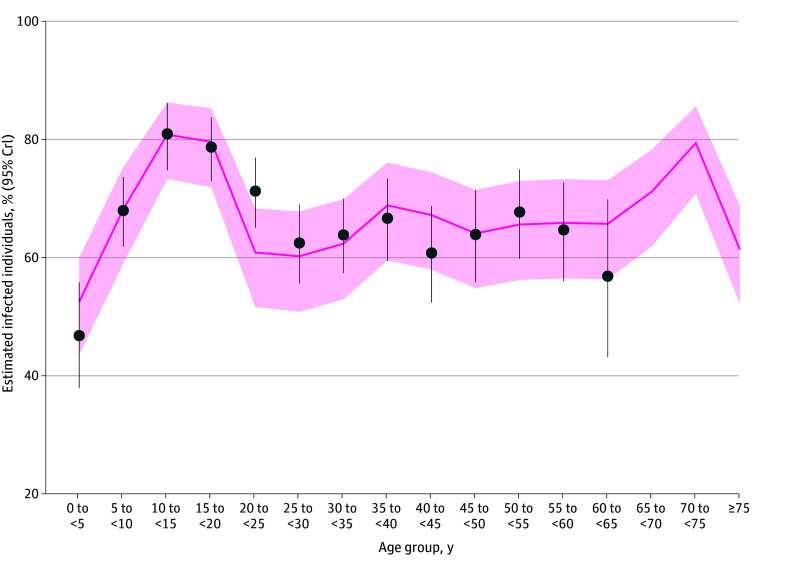
Estimated Infection Percentages for the 2014 to 2015 Chikungunya Virus Outbreak in French Polynesia, Using the Contact Matrix of France for Each 5-Year Age Group Dots show the observed postoutbreak seroprevalence for each 5-year age group; vertical bars indicate the associated 95% CI. The solid line shows the posterior mean model estimates, and shading indicates the associated 95% credible interval.

Results broadly aligned with the observed postoutbreak seroprevalence. Specifically, using the contact matrix of France, model estimates ranged from a minimum of 52.6% (95% CrI, 43.6%-60.1%) in those aged 0 to younger than 5 years to a maximum of 80.8% (95% CrI, 73.3%-86.2%) in those aged 10 to younger than 15 years (Figure 1); estimates for the other contact matrices are shown in eFigure 2 in Supplement 1.

#### R*_eff_* and Projections

After accounting for demographic characteristics and preexisting immunity in French Polynesia in 2025, all models produced varying estimates of R*_eff_* (eTable 2 in Supplement 1). Our best-fitting model, which utilized the contact matrix of France, yielded an R*_eff_* of 0.95 (95% CrI, 0.85-1.04). However, when assuming random mixing, the model estimated an R*_eff_* of 0.77 (95% CrI, 0.68-0.84).

**Figure 2.**
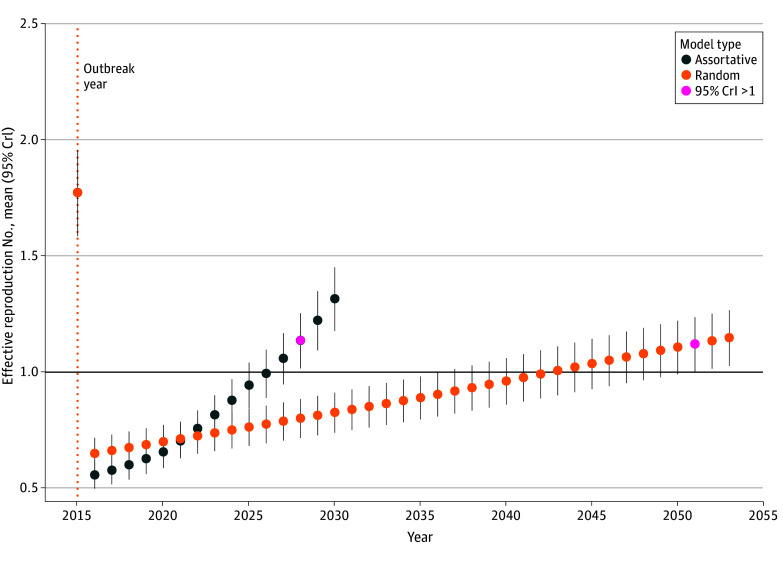
Projections of the Effective Reproduction Number Over Time Navy dots represent the mean effective reproduction number, while vertical bars indicate the associated 95% credible interval (CrI), assuming assortative mixing with the synthetic contact matrix for France. Orange dots represent the mean effective reproduction number, while vertical bars indicate the associated 95% CrI, assuming random mixing. The orange dotted vertical line marks the year of the first outbreak recorded and the solid horizontal line indicates the threshold for sustained transmission (effective reproduction number of 1). Red dots indicate the first year when the lower bound of the 95% CrI is greater than 1.

R*_eff_* projections showed that under assortative mixing (using the matrix of France), R*_eff_* would exceed 1 (ie, the lower bound of the 95% CrI would be greater than 1) by 2028 (Figure 2). In contrast, projections assuming random mixing showed that the outbreak threshold would be crossed starting in 2051.

## Discussion

French Polynesia has a history of arbovirus outbreaks triggered by introductions from other French overseas territories.^18,19,47^ The reemergence of CHIKV in La Réunion and Mayotte and autochthonous cases in metropolitan France^23,24,25,26^ prompted French Polynesia to perform a CHIKV outbreak risk assessment.

Analysis of data from a serosurvey conducted on adults between 2019 to 2021 showed that a decade after the last outbreak, with no CHIKV infection reported since, overall immunity against CHIKV remains high (67.6%). However, differences were observed.

Austral Islands residents exhibited significantly lower odds of CHIKV seropositivity than Windward Islands residents. This observation reflects surveillance system data, which reported fewer CHIKV infections in those islands during the 2014 to 2015 outbreak.^21^ Such spatial differences between subdivisions have also been documented for other arboviruses.^22,48^

Also, female participants exhibited higher odds of seropositivity than male participants. This sex discrepancy may be attributed to different social behaviors, as illustrated by contrasting findings from studies conducted in various regions worldwide.^49,50,51^ Additionally, age was significantly associated with CHIKV seropositivity. Seroprevalence peaked at nearly 81% in those aged 18 to 29 years, with higher odds of seropositivity than those in other age groups. Given no record of CHIKV circulation before the outbreak, all age groups were exposed simultaneously. Our findings suggest infection rates were higher among younger age groups during the outbreak, potentially reflecting age-related differences in social mixing patterns. This contrasts with dengue virus (DENV), which has caused multiple outbreaks over several decades, resulting in older age groups being more exposed on average and exhibiting higher seroprevalence.^52^

Examining additional sociodemographic factors, we found that higher education was associated with lower CHIKV seropositivity, consistent with previous studies in which lower educational attainment was associated with increased arbovirus prevalence.^53,54^ Although less documented,^55^ our study showed that unpartnered individuals had higher odds of seropositivity than never-married individuals. While the reasons for this association remain unclear, we hypothesize that marital status may act as a proxy for underlying factors influencing CHIKV exposure. Finally, living in a larger household was associated with higher odds of seropositivity, consistent with previous observations.^56^ This may reflect facilitated household-level transmission, as larger households offer more susceptible individuals when infected vectors are present.

Social mixing patterns are often incorporated into modeling human-to-human transmission of infectious diseases, such as influenza and COVID-19.^43,57^ Previous studies have demonstrated that human social structures and spatial proximity can affect CHIKV and DENV transmission.^27,28,29^ To understand how human social contacts could estimate age-specific risk for CHIKV, we used a model framework encompassing social contact matrices to estimate the 2014 to 2015 outbreak size, infer pre-outbreak R_0_, and assess reemergence risk. Our best-fitting model, which used the contact matrix for France, estimated an R_0_ of 1.78. This estimate aligns with previous findings that estimated an island-averaged R_0_ of 1.80 for French Polynesia, despite differences in model structure, input data, and the inclusion of climatic parameters.^58^ While comparable estimates were observed for the Dominican Republic,^59^ other outbreaks, such as in La Réunion Island^60^ and in the French Caribbean,^61^ exhibited higher R_0_ values. Interestingly, the Dominican Republic’s postoutbreak seroprevalence^59^ aligns with our findings, in contrast to La Réunion Island,^62^ Guadeloupe, and Martinique.^63^

By the end of the 2014 to 2015 outbreak in French Polynesia, health authorities estimated that approximately 69 000 individuals had been infected.^18,21^ Using France’s contact matrix as our primary proxy, we estimated that this number could have been doubled. Furthermore, we could derive age-specific infection levels, with highest estimates in preadolescents and younger adolescents. We hypothesize that the applicability of France’s contact matrix to our context stems from shared social characteristics, particularly the educational system, supported by studies indicating schools could represent a significant source of arboviral transmission.^64,65,66^

Another significant aspect of our study was to characterize CHIKV transmission and assess the risk of a new outbreak by the end of 2025. Assuming a scenario of lifelong CHIKV immunity, French Polynesia’s current age structure, and social mixing patterns equivalent to those of France, the R*_eff_* value was near the outbreak threshold (R*_eff_* >1), with credible intervals including values greater than 1. This suggests that localized transmission of CHIKV is likely to occur, particularly in areas with high vector density or lower population immunity. Moreover, R*_eff_* is expected to increase over time, thereby increasing the outbreak risk in coming years. When keeping all scenario parameters unchanged but assuming random mixing, we obtained lower R*_eff_* values, and the estimated timing of reemergence was delayed by approximately 20 years. Also, under random mixing, the observed adult seroprevalence (67.6%) would imply a slightly lower R_0_, which would further delay projected reemergence. This suggests that accounting for age-specific mixing might be important to avoid underestimating the risk of CHIKV reemergence.

### Limitations

While our approach provides novel insights, it has some limitations. The schoolchildren serosurvey was school based, relied on voluntary participation, and restricted to Tahiti, which may limit generalizability to all children in French Polynesia. In addition, because specific social mixing data for French Polynesia was unavailable, we used contact matrices from other countries as proxies. Additionally, we did not model variations in vector abundance and activity, which together with human mixing patterns can influence transmission risk.

## Conclusions

Our study, based on 2 cross-sectional serosurveys in French Polynesia, found that CHIKV seroprevalence was high a decade after the first outbreak, with differences between subdivisions, age groups, and other sociodemographic characteristics. We found that integrating age-specific social contact matrices reproduced the age-specific infection patterns during the 2014 to 2015 outbreak, underscoring the role of social contacts in CHIKV transmission. Additionally, results suggest that overlooking social contacts would underestimate reemergence timing. To our knowledge, this is among the first studies to use seroprevalence data and social mixing patterns to estimate age-specific past outbreak size, R_0_, and future reemergence risk for an arbovirus. Building on such an approach in other settings and for other arboviruses could enhance understanding of how age-specific immunity and social mixing patterns interact to shape arbovirus transmission dynamics.

## Notes

Supplement 1.
eMethods.
**eTable 1.** Basic Reproduction Number (R0) Estimates and Model Fit for French Polynesia Applying Empirical and Synthetic Contact Matrices **eTable 2.** Estimated Effective Reproduction Numbers (Reff) Based on Empirical and Synthetic Contact Matrices **eTable 3.** Risk Factors Associated With CHIKV Seropositivity in Full Univariate and Multivariable Logistic Regression Models **eFigure 1.** Infection Rate Estimates per Age Group for the 2014-2015 CHIKV Outbreak (Empirical Matrices) **eFigure 2.** Infection Rate Estimates per Age Group for the 2014-2015 CHIKV Outbreak (Synthetic Matrices) **eFigure 3.** Bootstrap Distribution of Basic Reproduction Number (R_0_) Estimates for Country-Specific Contact Matrix **eFigure 4.** Bootstrap Distribution of Maximized Log-Likelihood for Country-Specific Contact Matrix

Supplement 2.
Data Sharing Statement
