# Supplementary material for: Social Mixing Patterns and Chikungunya Reemergence Risk in French Polynesia
Source: JAMA Netw Open. 2026 Mar 18;9(3):e262270. doi: 10.1001/jamanetworkopen.2026.2270 (PMC13000641; doi:10.1001/jamanetworkopen.2026.2270)
Supplement: Supplement 1. — eMethods. eTable 1. Basic Reproduction Number (R0) Estimates and Model Fit for French Polynesia Applying Empirical and Synthetic Contact Matrices eTable 2. Estimated Effective Reproduction Numbers (Reff) Based on Empirical and Synthetic Contact Matrices eTable 3. Risk Factors Associated With CHIKV Seropositivity in Full Univariate and Multivariable Logistic Regression Models eFigure 1. Infection Rate Estimates per Age Group for the 2014-2015 CHIKV Outbreak (Empirical Matrices) eFigure 2. Infection Rate Estimates per Age Group for the 2014-2015 CHIKV Outbreak (Synthetic Matrices) eFigure 3. Bootstrap Distribution of Basic Reproduction Number (R0) Estimates for Country-Specific Contact Matrix eFigure 4. Bootstrap Distribution of Maximized Log-Likelihood for Country-Specific Contact Matrix [file jamanetwopen-e262270-s001.pdf]

Supplemental Online Content

Chung K, Aubry M, Teiti I, et al. Social mixing patterns and chikungunya reemergence risk in French Polynesia. *JAMA Netw Open.* 2026;9(3):e262270. doi:10.1001/jamanetworkopen.2026.2270

eMethods.

eTable 1. Basic Reproduction Number (R0) Estimates and Model Fit for French Polynesia Applying Empirical and Synthetic Contact Matrices

eTable 2. Estimated Effective Reproduction Numbers (Reff) Based on Empirical and Synthetic Contact Matrices

eTable 3. Risk Factors Associated With CHIKV Seropositivity in Full Univariate and Multivariable Logistic Regression Models

eFigure 1. Infection Rate Estimates per Age Group for the 2014-2015 CHIKV Outbreak (Empirical Matrices)

eFigure 2. Infection Rate Estimates per Age Group for the 2014-2015 CHIKV Outbreak (Synthetic Matrices)

eFigure 3. Bootstrap Distribution of Basic Reproduction Number (R_0) Estimates for Country-Specific Contact Matrix

eFigure 4. Bootstrap Distribution of Maximized Log-Likelihood for Country-Specific Contact Matrix

This supplemental material has been provided by the authors to give readers additional information about their work.

© 2026 Chung K et al. *JAMA Network Open*.

eMethods

Model comparison

Since all models had the same number of parameters (k=1), model comparison was performed by evaluating the maximized log-likelihood ($\log(L_{MLE})$) using a Bernoulli likelihood function that compared observed and predicted infection probabilities for each model. As a higher value indicates a better fit of the model to the observed data, the model with the highest value was chosen as the preferred model. The difference between the maximized log-likelihood values ($\Delta\log(L_{MLE})$) was calculated for each model relative to the highest value.

Sensitivity analysis

We also assessed the sensitivity of R_0 to variations in the serological data in addition to the choice of contact matrix. For that, we generated 100 bootstrap resamples of the combined serological data and inferred the different R_0 by running 2,000 iterations after an initial burn-in of 500 for each matrix (eFigure 3). We also computed the maximized log-likelihood ($\log(L_{MLE})$) for every bootstrap replicate and compared these values with the value obtained from the original serological dataset to evaluate whether the model delivered consistent conclusions irrespective of the input data (eFigure 4).

eTable 1. Basic Reproduction Number (R_0) Estimates and Model Fit for French Polynesia Applying Empirical and Synthetic Contact Matrices

Country matrix	Estimated R_0 [95%Credible Interval]	$log(L_{MLE})$	$\Delta log(L_{MLE})$
Empirical			
**Belgium**	1.71 [1.67 - 1.75]	-1501	-29
**Finland**	1.75 [1.70 - 1.79]	-1497	-25
**Germany**	1.72 [1.67 - 1.76]	-1515	-43
**Italy**	1.85 [1.81 - 1.90]	-1488	-16
**Luxembourg**	1.74 [1.70 - 1.78]	-1494	-22
**Netherlands**	1.78 [1.74 - 1.83]	-1527	-55
**Poland**	1.79 [1.74 - 1.83]	-1496	-24
**United Kingdom**	1.73 [1.68 - 1.77]	-1480	-8
Synthetic			
**Fiji**	1.80 [1.75 - 1.84]	-1473	-1
**France**	**1.78 [1.73 - 1.82]**	**-1472**	**Ref.**
**Samoa**	1.88 [1.84 - 1.94]	-1492	-20
**Solomon Islands**	1.73 [1.68 - 1.77]	-1479	-7
**Tonga**	1.87 [1.82 - 1.92]	-1479	-7
**Vanuatu**	1.77 [1.72 - 1.81]	-1481	-9

R_0, basic reproduction number; $log(L_{MLE})$, maximized log-likelihood; $\Delta log(L_{MLE})$difference in maximized log-likelihood from the preferred model; Ref., reference

© 2026 Chung K et al. *JAMA Network Open*.

eTable 2. Estimated Effective Reproduction Numbers (R_{eff}) Based on Empirical and Synthetic Contact Matrices

Country matrix	Estimated R_{eff} – *assortative mixing* [95% CrI]	Estimated R_{eff} – *random mixing* [95% CrI]
Empirical		
**Belgium**	0.72 [0.63 – 0.79]	0.74 [0.65 – 0.82]
**Finland**	0.96 [0.86 – 1.06]	0.75 [0.67 – 0.84]
**Germany**	0.75 [0.67 – 0.83]	0.74 [0.66 – 0.82]
**Italy**	0.93 [0.84 – 1.02]	0.80 [0.71 – 0.88]
**Luxembourg**	0.93 [0.83 – 1.03]	0.75 [0.67 – 0.84]
**Netherlands**	1.37 [1.22 – 1.51]	0.77 [0.68 – 0.85]
**Poland**	0.83 [0.74 – 0.91]	0.78 [0.70 – 0.86]
**United Kingdom**	1.06 [0.94 – 1.17]	0.74 [0.66 – 0.82]
Synthetic		
**Fiji**	0.96 [0.86 – 1.06]	0.77 [0.69 – 0.85]
**France**	**0.95 [0.85 – 1.04]**	**0.77 [0.68 – 0.84]**
**Samoa**	1.12 [1.00 – 1.22]	0.81 [0.73 – 0.89]
**Solomon Islands**	0.98 [0.87 – 1.08]	0.74 [0.66 – 0.82]
**Tonga**	1.01 [0.91 – 1.11]	0.80 [0.72 – 0.89]
**Vanuatu**	1.01 [0.90 – 1.11]	0.76 [0.68 – 0.84]

R_{eff}, effective reproduction number; CrI, credible interval

© 2026 Chung K et al. *JAMA Network Open*.

eTable 3. Risk Factors Associated With CHIKV Seropositivity in Full Univariate and Multivariable Logistic Regression Models

	Univariate model		Multivariate model	
	OR[1](95% CI)	p-value	aOR[1](95% CI)	p-value
**Sex**		0.013		**0.010**
Female	—		—	
Male	0.73* (0.56, 0.94)	0.013	0.71* (0.54, 0.92	0.010
**Age group**		<0.001		**<0.001**
18-29 years	—		—	
30-44 years	0.45*** (0.32, 0.63)	<0.001	0.47*** (0.32, 0.67)	<0.001
45-69 years	0.35*** (0.25, 0.49)	<0.001	0.33*** (0.22, 0.49)	<0.001
**Highest education level**		0.001		**0.002**
End of primary or before	—		—	
End of secondary school	1.10 (0.73, 1.64)	0.65	0.93 (0.61, 1.43)	0.74
End of high school or equivalent	1.32 (0.90, 1.94)	0.16	0.96 (0.63, 1.48)	0.87
University or after	0.67 (0.45, 1.01)	0.05	0.51** (0.32, 0.81)	0.005
**Marital status**		0.086		**0.031**
Never married	—		—	
In a relationship	0.78 (0.58, 1.05)	0.10	0.91 (0.65, 1.27)	0.56
Unpartnered	1.25 (0.70, 2.23)	0.45	1.96* (1.03, 3.72)	0.039
**Subdivision**		<0.001		**<0.001**
Windward Islands	—		—	
Leeward Islands	1.40** (1.09, 1.80)	0.009	1.34* (1.02, 1.76)	0.033
Marquesas Islands	1.15 (0.82, 1.61)	0.41	1.25 (0.87, 1.80)	0.23
Tuamotu-Gambier Islands	1.67** (1.17, 2.38)	0.005	1.69** (1.15, 2.50)	0.008
Austral Islands	0.42*** (0.31, 0.57)	<0.001	0.39*** (0.28, 0.56)	<0.001
**Household size**		<0.001		**0.048**
1-2	—		—	
3-5	1.38* (1.01, 1.89)	0.044	1.17 (0.83, 1.65)	0.36
6+	2.27*** (1.55, 3.32)	<0.001	1.66* (1.09, 2.52)	0.018
**House type**		0.066		0.20
House without garden	—		—	
House with garden	0.60 (0.34, 1.03)	0.066	0.69 (0.39,1.23)	0.20
**Air conditioning**		0.21		
Yes	—			
No	1.21 (0.90, 1.63)	0.21		
**Clean water access**		0.21		
Yes	—			
No	1.43 (0.82, 2.51)	0.21		
**Mosquito bites frequency**		0.43		
Never	—			
Rarely	0.66 (0.29, 1.52)	0.32		
Often	0.77 (0.33, 1.80)	0.55		
Everyday	0.82 (0.36, 1.89)	0.64		

© 2026 Chung K et al. *JAMA Network Open*.

eTable 3. Risk Factors Associated With CHIKV Seropositivity in Full Univariate and Multivariable Logistic Regression Models

	Univariate model		Multivariate model	
	**OR[1](95% CI)**	**p-value**	**aOR[1](95% CI)**	**p-value**
**Number of mosquito protection used**		0.83		
0	—			
1-2	0.99 (0.54, 1.82)	0.98		
3-4	0.95 (0.52, 1.73)	0.85		
5+	0.83 (0.43, 1.60)	0.57		

[1] **p<0.05; **p<0.01; ***p<0.001; OR, Odds Ratio; aOR, Adjusted Odds Ratio; CI, Confidence Interval*

© 2026 Chung K et al. *JAMA Network Open*.

eFigure 1. Infection Rate Estimates per Age Group for the 2014-2015 CHIKV Outbreak (Empirical Matrices)

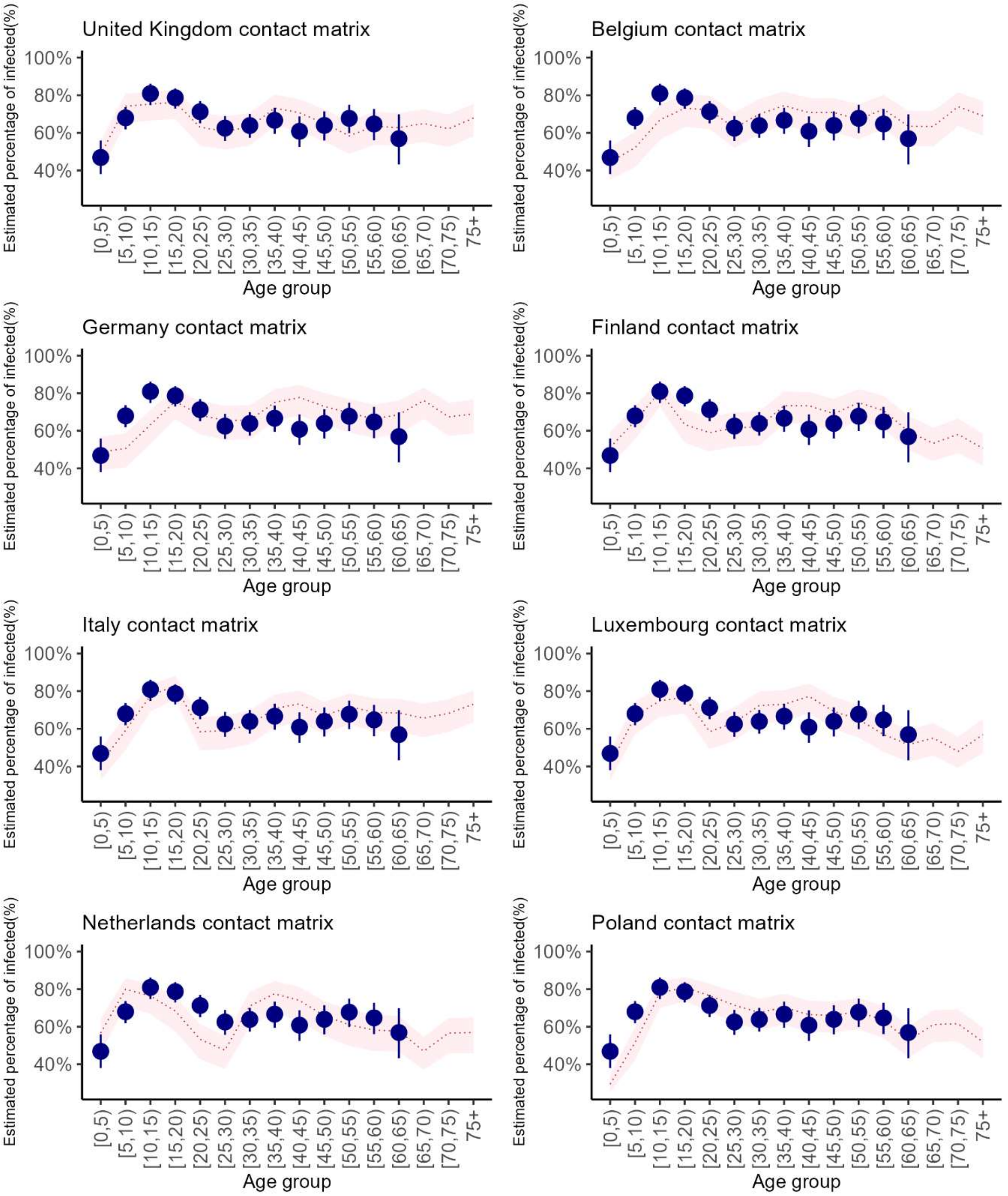

Navy dots show the observed post-outbreak seroprevalence for each five-year age group; navy vertical bars indicate the associated 95%C*I*; red dotted line show the posterior mean model estimates, and pink shading indicate the associated 95%CrI.

© 2026 Chung K et al. *JAMA Network Open*.

eFigure 2. Infection Rate Estimates per Age Group for the 2014-2015 CHIKV Outbreak (Synthetic Matrices)

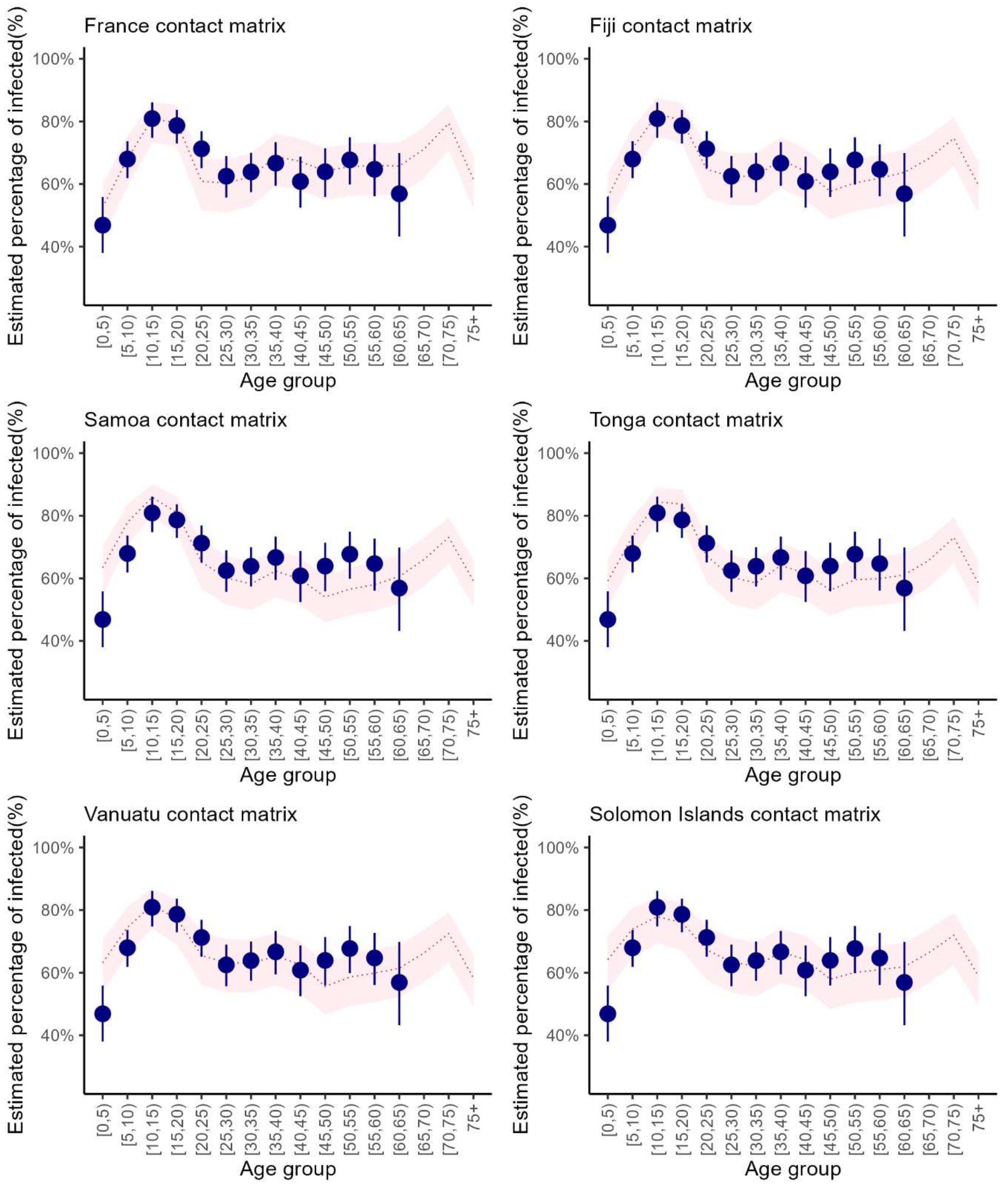

Navy dots show the observed post-outbreak seroprevalence for each five-year age group; navy vertical bars indicate the associated 95%CI; red dotted line show the posterior mean model estimates, and pink shading indicate the associated 95%CrI.

© 2026 Chung K et al. *JAMA Network Open*.

eFigure 3. Bootstrap Distribution of Basic Reproduction Number (R_0) Estimates for Country-Specific Contact Matrix

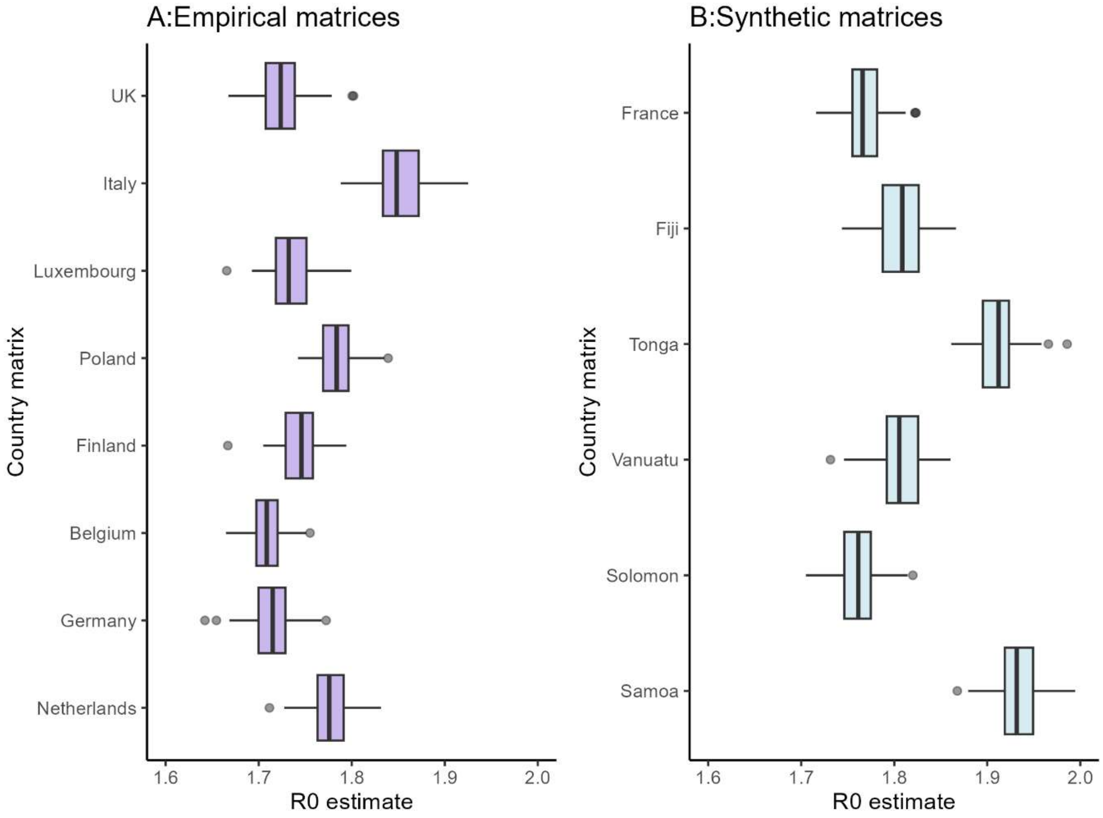

Boxplots summarise R_0 estimates across bootstrap replicates (centre line= median; box= interquartile range; points=outliers). Panel A (purple) shows values per country for empirical (POLYMOD study) matrices and Panel B (light blue) for synthetic (France and five Pacific Islands countries) matrices.

© 2026 Chung K et al. *JAMA Network Open*.

eFigure 4. Bootstrap Distribution of Maximized Log-Likelihood for Country-Specific Contact Matrix

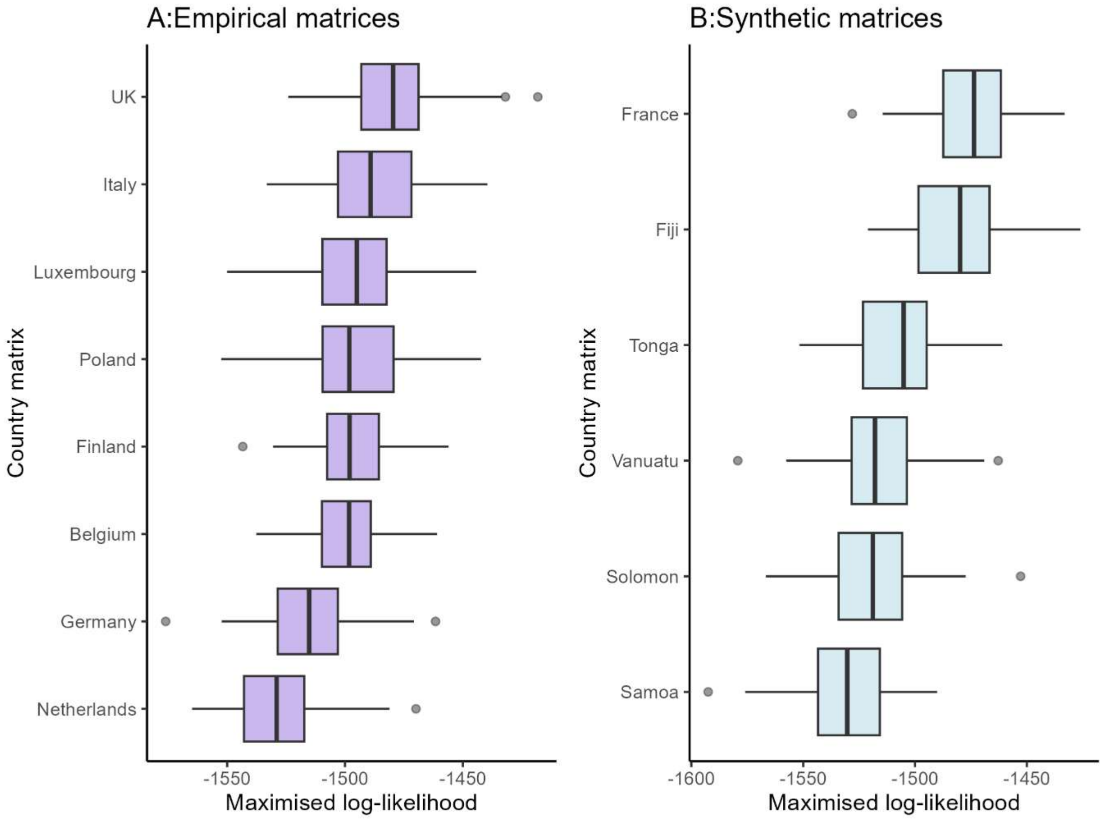

Boxplots summarise maximized log-likelihood values across bootstrap replicates with higher values indicating better fit (centre line= median; box= interquartile range; points= outliers). Panel A (purple) shows values per country for empirical (POLYMOD study) matrices and Panel B (light blue) for synthetic (France and five Pacific Islands countries) matrices.

© 2026 Chung K et al. *JAMA Network Open*.
