# Supplementary material for: Social Mixing Patterns and Chikungunya Reemergence Risk in French Polynesia
Source: JAMA Netw Open. 2026 Mar 18;9(3):e262270. doi: 10.1001/jamanetworkopen.2026.2270 (PMC13000641; doi:10.1001/jamanetworkopen.2026.2270)
Supplement: Supplement 2. — Data Sharing Statement [file jamanetwopen-e262270-s002.pdf]

Data Sharing Statement

Chung. Social Mixing Patterns and Chikungunya Reemergence Risk in French Polynesia.
JAMA Netw Open. Published March 18, 2026. doi:10.1001/jamanetworkopen.2026.2270

Data
Data available: Yes
Data types: Deidentified participant data
How to access data: GitHub (https://github.com/kiyochung/chikungunya-reemergence)
When available: With publication

Supporting Documents
Document types: Statistical/analytic code
How to access documents: GitHub (https://github.com/kiyochung/chikungunya-reemergence)
When available: With publication

Additional Information
Who can access the data: Anyone who wishes to access the data
Types of analyses: To reproduce key figures in the paper
Mechanisms of data availability: Data and code will be available following publication.
